# Investigation on CT characterization of pore structure in nylon-uncured rubber composite from a microscopic view

**DOI:** 10.1038/s41598-021-95178-1

**Published:** 2021-08-03

**Authors:** Yong Li, Yanmeng Chi, Shanling Han, Yanan Miao, Long Chen

**Affiliations:** 1 College of Mechanical and Electronic Engineering, Shandong University of Science and Technology, Qingdao, 266590 China; 2 College of Safety and Environmental Engineering, Shandong University of Science and Technology, Qingdao, 266590 China; 3 College of Materials Science and Engineering, Shandong University of Science and Technology, Qingdao, 266590 China

**Keywords:** Composites, Mechanical properties

## Abstract

In order to construct the geometric models characterizing the real micro pore-fracture structures of nylon-uncured rubber composite, and further compare the distribution law in the pore-fracture of solid (nylon)-gas (pore) two-phases with that of solid (nylon)-viscoelastic body (rubber)-gas (pore) three-phases composite, in this paper, the X-ray three-dimensional (3D) microscope is applied for the nylon material and nylon-rubber composite respectively. By employing the 3D visualization software (Avizo), three-dimensional reconstruction and pore-fracture network model is realized, where the quantitative statistics and comparative analysis are carried out. The results demonstrate that the pore/throat number of nylon material accounting for 20.8%/33.9% are the largest when the pore/throat radius is in the range of 3–4 μm/1–2 μm, respectively, however, the pore/throat number of nylon-rubber composite with the radius 3–4 μm/1–2 μm occupies merely 5.49%/11.3%. Furthermore, the average pore radius of nylon material is believed as larger than that of nylon-rubber composite based on the pore network model, where the pore/throat surface area and pore/throat volume have perfect consistent patterns with that of pore radius. This work will offer a theoretical basis for the investigation of gas seepage capability discrepancy between the solid (nylon) one-phase and solid (nylon)-viscoelastic body (rubber) two-phases.

## Introduction

Vulcanized nylon-rubber composite, with its excellent mechanical properties, has become the preferred skeleton of rubber industry, which is available to be applied in large rubber products, such as tires, air springs, transport belts, sealing rings, etc. Researchers possess good ability to design products with different nylon-rubber structures depending on their subjective wishes, but the actual performance is far from the desired one. For example, among the 1718 cases of tire recall in the United States published by the National Highway Traffic Safety Administration, 127 cases belong to the top five brands in the world^1^, and the direct correlation to fiber-rubber composite (lamination and interlayer lack of glue) accounts for 49.6%.

As a safety product, tire is the only grounding part of automobile. Nylon-rubber composite plays a key role in tire body, belt layer and other parts. The research on nylon-rubber becomes a golden key to improve tire quality, which is not available to be ignored by the top tire companies, but why the frequent recall incidents occur? Firstly, the company's process control is in place to ensure the product consistency to the greatest extent; Secondly, the indirect quality testing of uncured nylon-rubber composite materials is carried out depending on the previous experience, which is emphasized on manufacturing results and lack of process control. For example, by employing the vulcanized cord extraction force and hang rubber amount to indirectly determine the degree of rubber penetration in cord. Thirdly, the research on the solution of fiber-rubber delamination focuses on the macroscopic mechanical research. The complex cord structure is equivalent to a rebar and the interface layer is idealized, lacking the research on its "birth process" at a mesoscopic scale.

**Figure 1:**
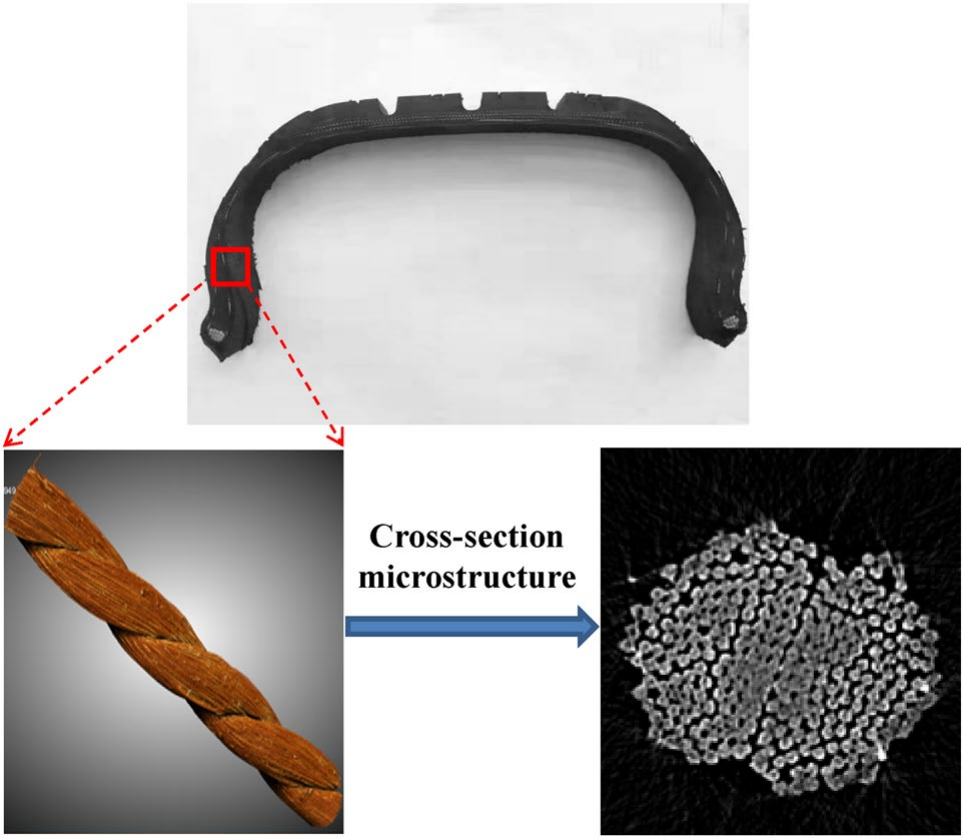
Radial tire structure diagram^2^.

The nylon structure is composed of massive single filaments, each of which is a spring, twisted in different arrangements, as shown in Fig. 1. Therefore, under the condition of ignoring contact stress, nylon as a whole will present the viscoelasticity; In addition, in order to improve the cohesive performance, nylon is available to be utilized as a porous viscoelastic skeleton material. According to the mesoscopic structure of nylon, it can be regarded as a dual medium composed of pore and fracture: pores are formed by single strand, which is manifested as interstice or linear contact between nylon; fractures are formed by single filaments and appear as point contact.

**Figure 2:**
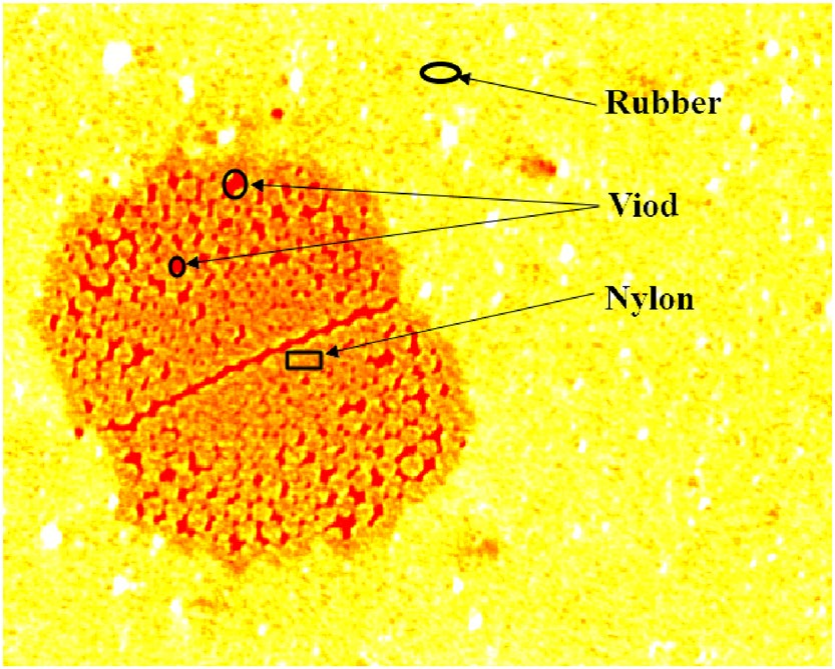
Microstructure of rubber, nylon and air in composites.

The important mechanism of rubber-nylon delamination is the thermal oxygen aging of rubber in the interface. It should be noted from Fig. 2 that the distribution of uncured rubber-nylon-void (air) and their interactions will determine the material distribution, interface strength, thermal and oxygen aging of the made-up products, as well as their performance. The effective measure is to reduce the content of air on the interface, which is necessary to decrease the invalid gap. At present, the common macroscopic indirect means are utilized to determine the performance of nylon percolating, which lacks direct quantitative analysis of nylon percolating in the fine structure. Therefore, it is a vital attempt to study the rubber permeability based on the percolation theory^3–7^, by regarding the nylon as porous media and melt rubber gradually permeating into nylon then removing air. By employing the micro Computed tomography (CT) technology to realize the pore-fracture study of above-mentioned distribution and evolution, it is a key technique to establish the coupling nylon-rubber percolation model. As a result, it is particularly significant to accurately characterize the structure and morphology of nylon-rubber pore and fracture^8,9^. Uncured nylon-rubber material has not been cross-linked, as well the tensile and bonding strength are low, resulting in that the conventional means are relatively difficult to be employed.

**Figure 3:**
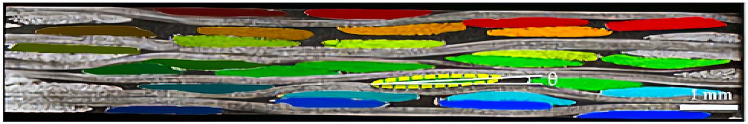
MicroCT image of polyester fabric^5^.

Till now, the research about nylon is mainly limited to the engineering mechanical properties, such as elongation at break or twist, but lacks the discussion on 3D fine structure and constitutive model of nylon. Currently, the research on mesoscopic structure of 3D fabric can provide useful reference^10–12^, focusing on three aspects: (1) Different simplified fine structures are adopted, such as: conformal finite element mesh^13^, multielement digital chain^14^, discrete element method^15^ and structure genome^16^; (2) Direct detection methods such as micro-CT^17^ (Fig. 3) and X-ray tomography^11^ are used to determine the real geometric model of 3D composite reinforced materials, and mesoscopic finite element simulation is used to acquire the mechanical properties and air permeability of composite materials; (3) Based on the mechanics of monofilaments continuum^18^ and the characterization of mesoscopic mechanics of volume elements^19^, a constitutive model based on the principle of multi-scale structure is established to reflect the microstructure changes of three-dimensional textile composites. The mesoscopic model of nylon fine structure in uncured nylon–rubber material has not been studied so far.

Owing to the fiber structure, small rubber molecules and assembly process, some air or moisture are inevitably existed in the rubber-nylon composites. Based on the currently-reported literatures, engineers mainly employ the indirect methods to control the product quality, such as measuring the air content in nylon or extraction force of nylon, but the exact distribution of nylon, rubber and air remains ambiguous. In this paper, the CT scanning technology is employed to test the fine micro-structure of nylon material and nylon-uncured rubber composites, respectively. In addition, the three-dimensional reconstruction is conducted by employing the 3D visualization software “Avizo” (9.5 Version). Furthermore, the interception of materials is realized to acquire the quantitative statistics of nylon material and nylon-uncured rubber composite, such as microscopic parameters (pore size, pore-fracture characteristics). This work that testing the void distribution and evolution is a key basis on establishing the coupling penetration models, which is an important attempt to further investigate the rubber permeability.

## Materials and processing

The nylon-uncured rubber composite studied in this paper is made up of rubber compound and nylon 66 (polyadiohexylenediamine). The sample ingredients utilized in this test comprised natural rubber (NR), carbon black (CB) N326, aromatic oil, stearic acid, sulfuric agent, accelerator (DZ) and anti-aging agent. Details are provided in Table 1.

**Table 1:** Amounts of main ingredients in the sample.

Ingredient	Amount (phr)	Ingredient	Amount (phr)
Natural rubber (NR)	100	Stearic acid	1.5
Carbon black (CB) N326	45	Sulfuric agent	3
Aromatic oil	3	Accelerator (DZ)	1
ZnO	5	Anti-aging agent	2.5

To obtain the stable and reliable samples, the uncured rubber compound is required to be treated in two steps before the experiment. Firstly, three-stage mixing takes place in a mixer; Then, the rubber compound is softened to the viscous state by a cold feed extruder to keep the temperature at 95 ± 5 °C in preparation for the combination of rubber and fiber.

Nylon 66 is characterized by high breaking strength, wear resistance, fatigue resistance, impact resistance, good dimensional stability and strong adhesive force with rubber. It is the main industrial materials for airbags, rubber/fiber composites and conveyer belts, etc. Nylon 66 is polycondensation from hexylenediamine adipate, with the composition of HO-[OC(CH2)4COHN (CH2)6NH]n–H. The type adopted in this experiment is 930Dtex/2, produced by Shenma Group Co., LTD. It is made up of two single filaments, with twist (T/10 cm) 46.0 ± 1.5, diameter 0.55 ± 0.03 mm, dip pick-up 4.5 ± 0.9% and shape coefficient 0.92.

**Figure 4:**
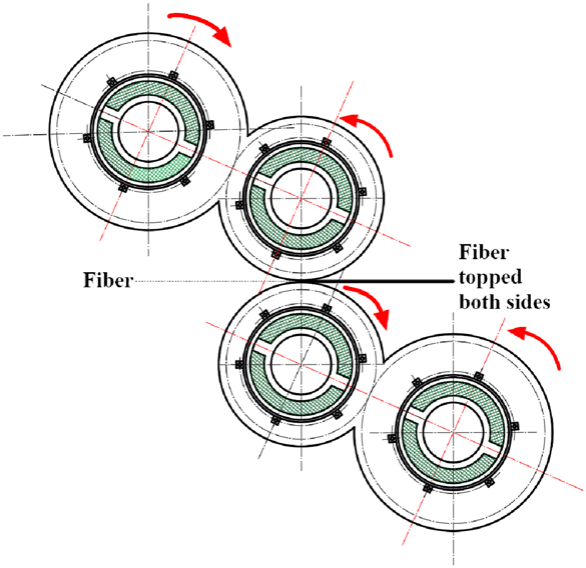
The S-type four-roll calender.

Then, the sample is manufactured by the S-type four-roll calender (Fig. 4). Calendering is one of the two most important processes used to combine fibers and rubber compound, which the rubber sheet is presented to the fabric and pressed onto the top of the fabric. It is necessary for nylon fabrics, to be dried, prior to entry into the calender nip. Usually, this is achieved by passing the fabric over a set of rolls, at temperatures of around 100–110 °C. The rubber stock is passed through a series of gaps of decreasing size made by a stand of rotating rolls and the rubber sheet thickness was determined by final roll gap. Both sides of the fabric can be topped on one pass. During the assembly, it is critical to avoid any entrapment of air or volatile materials. To avoid air bubbles, it is a fairly common practice to use a spiked roller to allow egress of air or to use a profiled compaction roller, with a slight bow in the center, to push any air to the outside of the composite being assembled.

**Figure 5:**
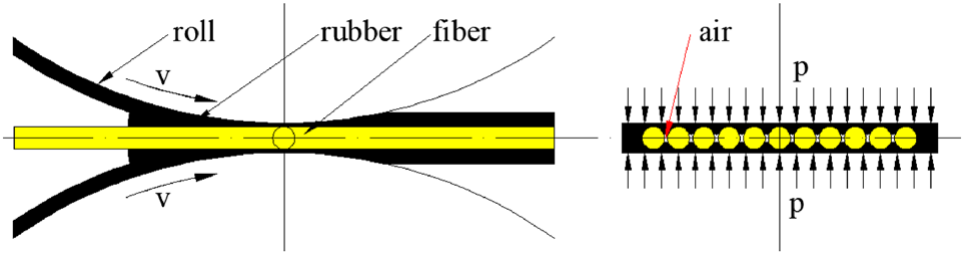
Top the rubber compound onto both faces of the fiber, with some air contained in the rubber/fiber composite.

However, some air or moisture will always remain in the rubber/cord composite, owing to the fiber structure, small molecules in rubber and the assembly processing (Fig. 5). At present, indirect methods such as measuring cord air content or cord extraction force are mainly used to control product quality, but the exact distribution of cord, rubber and air is not known.

## Quantitative characterization of pore-fracture structure in the nylon material

### CT scan

A 3D nondestructive and high-resolution X-ray microscope (nanoVoxel-3000) is utilized to characterize the internal pore structure of nylon from a microscopic scale. Combined with the Avizo software, the statistical calculation of three-dimensional pore connectivity, porosity, throat size and various seepage characteristic parameters in the internal pore-throat of nylon is realized.

**Figure 6:**
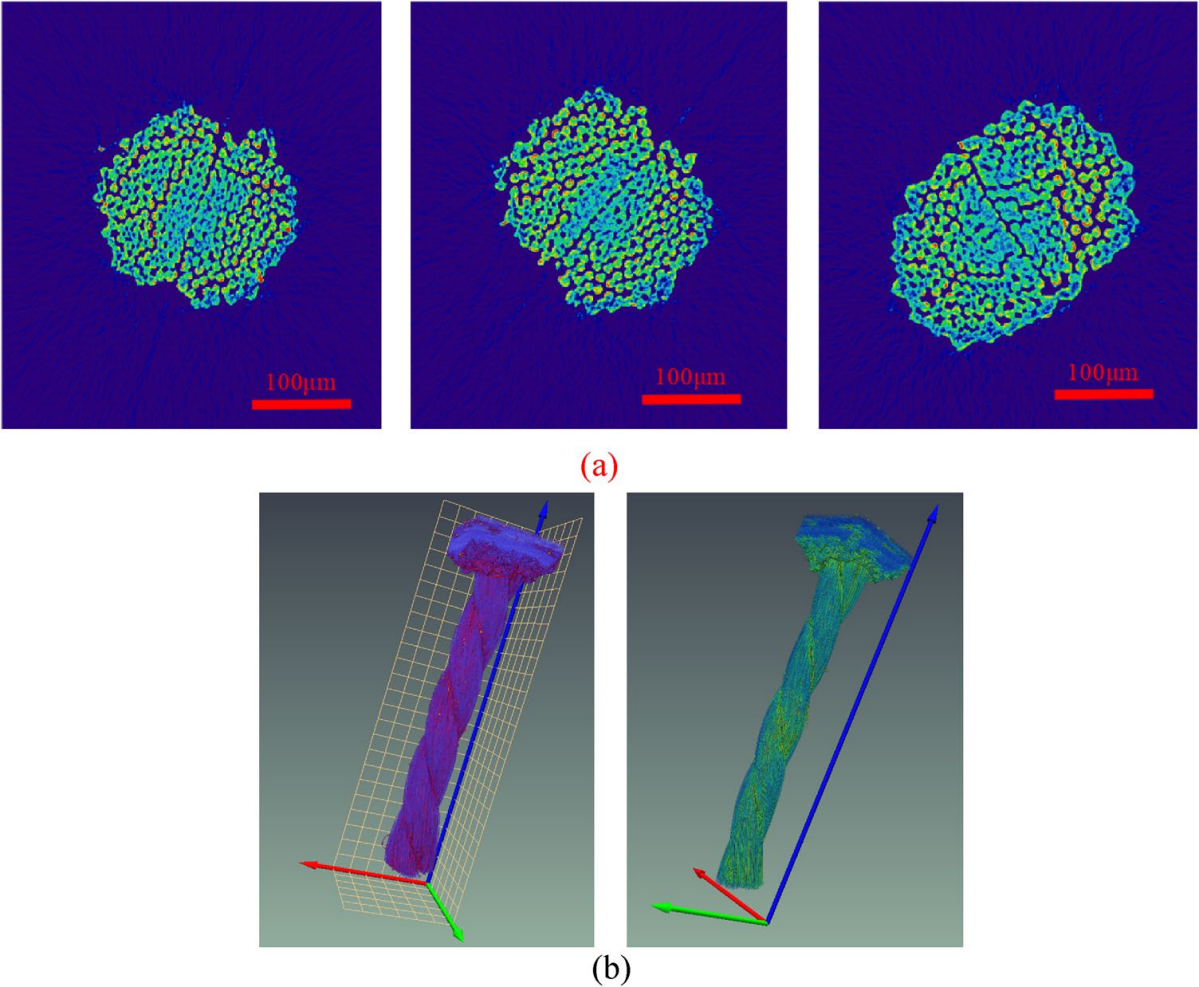
The layered scan and three-dimensional images of nylon.

The selected nylon samples with a size of 500 × 500 × 2272 are put into the X-ray 3D microscope scanning table. In order to ensure the scanning accuracy, the nylon is fixed as far as possible during the rotating process of the sample table. The setting of CT scanning parameters is presented in Table 2. The layered scan images of nylon are indicated in Fig. 6a, and the three-dimensional images of nylon are described in Fig. 6b.

**Table 2:** Scanning parameters of X-ray 3D microscope.

Detector type	SOD (MM)	Voltage (kV)	Current (µA)	Scanning frame number time (s)	Time (h)	Exposure time (s)	Penetration rate (%)	SDD (MM)
Tablet 2940*2304	21.3073	90	60	1440	2	0.4	90	371.1715

### CT three-dimensional reconstruction

A large number of two-dimensional nylon images are obtained through the CT scanning technology. In order to ensure that the digital model could truly reflect the actual pore structure characteristics of nylon, the elliptical sections were selected from the middle part of nylon scanning image for three-dimensional reconstruction.

**Figure 7:**
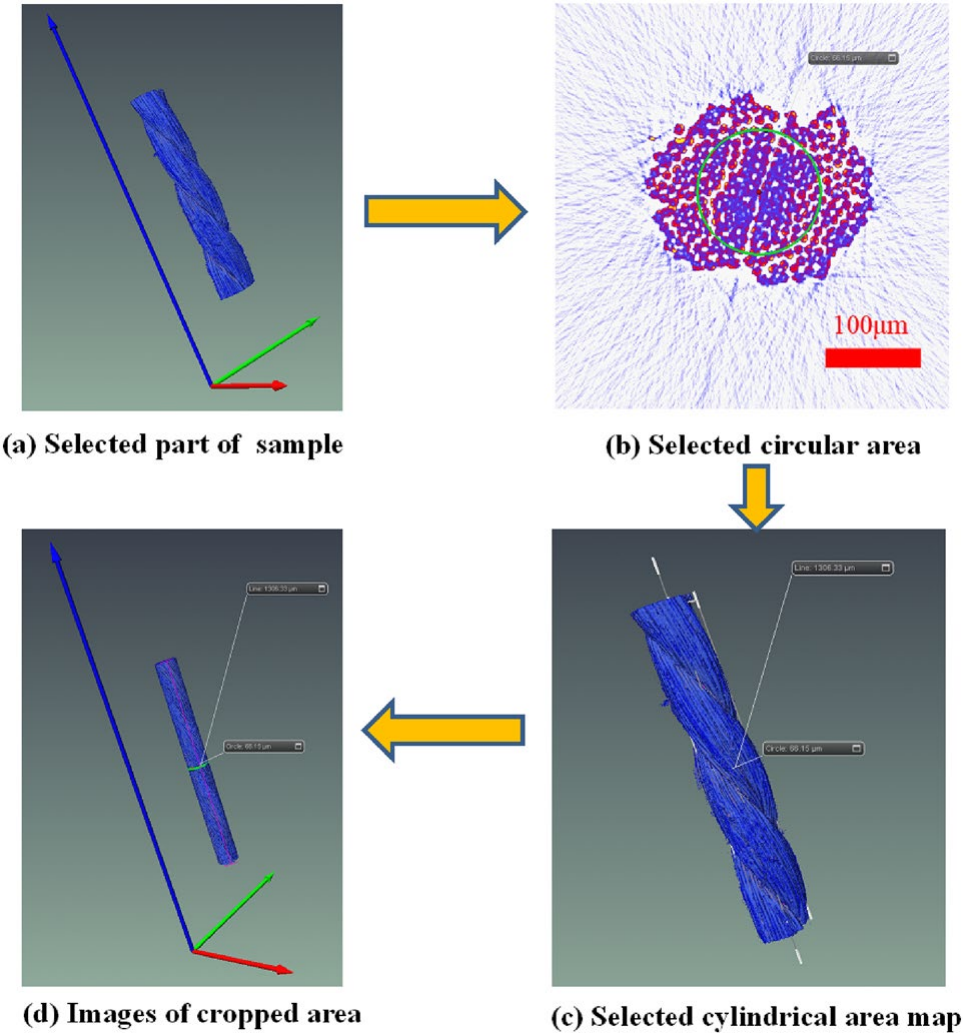
Area selection from the nylon CT images.

**Figure 8:**
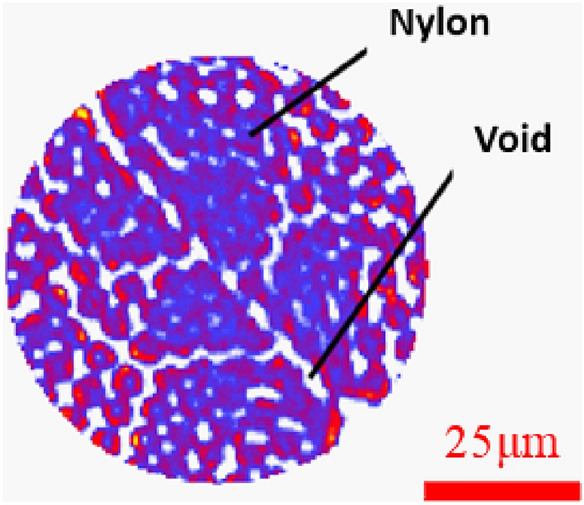
The division diagram of nylon and void (the purple part is nylon, and the white part inside is pore).

A continuous 2271 images were adopted from the nylon CT images obtained by scanning. Due to the good quality of original data, no filtering was needed, and the sections could be directly reconstructed in 3D. In the data volume, the center point of a nylon slice was used as a cylinder with a radius of 66.15 μm and a height of 1306.33 μm to frame out the study area, which is illustrated in Fig. 7. Figure 7a indicates that part of the sample cut from the nylon sample. Figure 7(b) describes the circular area selected from the section. Figure 7c shows the pattern when cutting with the cylinder. Figure 7d illustrates the enlarged area after cutting. It should be noted that a section was selected to obtain the differentiation diagram of the void(air) and nylon material, as shown in Fig. 8.

Through the 3D reconstruction technology, the real data model of nylon can be acquired by stacking the two-dimensional slices obtained by scanning, that is, the research objects are basically the same in size. The establishment of data volume model is the foundation for the analysis of pore-fracture structure parameters.

### Construction of pore-fracture structure model

**Figure 9:**
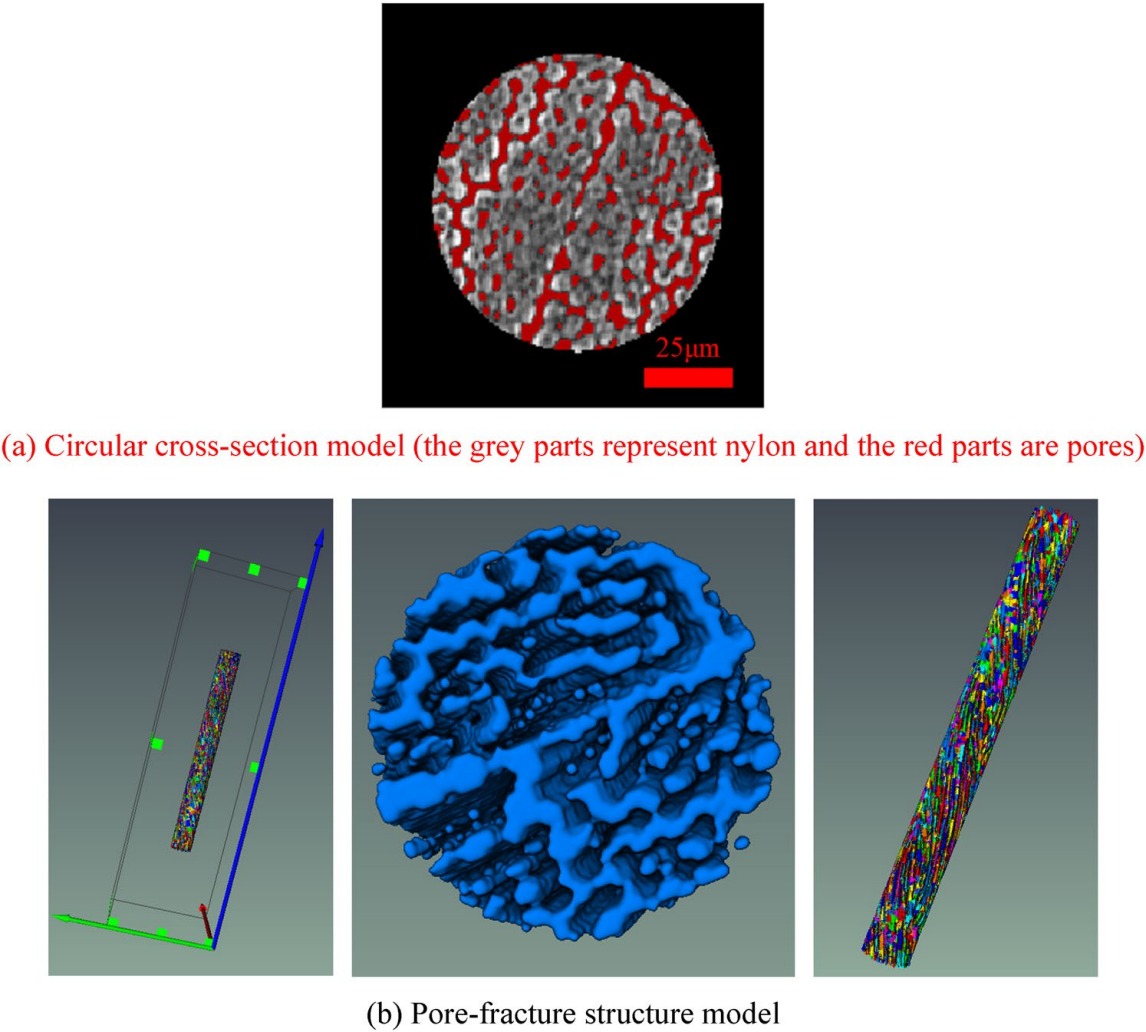
Three-dimensional distribution of the model.

On the basis of the three-dimensional entity obtained by the threshold segmentation, the pore and fracture structure are available to be extracted, as indicated in Fig. 9. In the process of constructing the pore-fracture structure model, owing to the clear slice color and the large degree of differentiation, the “Interactive Thresholding Command” in Avizo is adopted for image segmentation. This is necessary to select the threshold value of the pore-fracture structure, and the acquired overall porosity should not be too small to ensure that the unconnected pores are eliminated. The effective porosity is in line with the true porosity of nylon, seen as the red part of Fig. 9a. Figure 9b demonstrates a pore-fracture structure model, which can be acquired that the pores are irregularly dotted and the interconnected throat shapes. Through the analysis of pore-fracture structure model, the porosity of nylon sample yields 0.241578, and further the detailed quantitative parameters of pore model are obtained, as indicated in Table 3.

**Table 3:** Quantitative parameter statistics of pore model in nylon material.

Parameters	Pore radius (μm)	Pore volume (μm^3^)	Pore surface area (μm^2^)
Maximum	100.1005	4,201,420	3,061,350
Average	1.234675	853.476	646.078
Minimum	0.62035	1	3.00419

**Figure 10:**
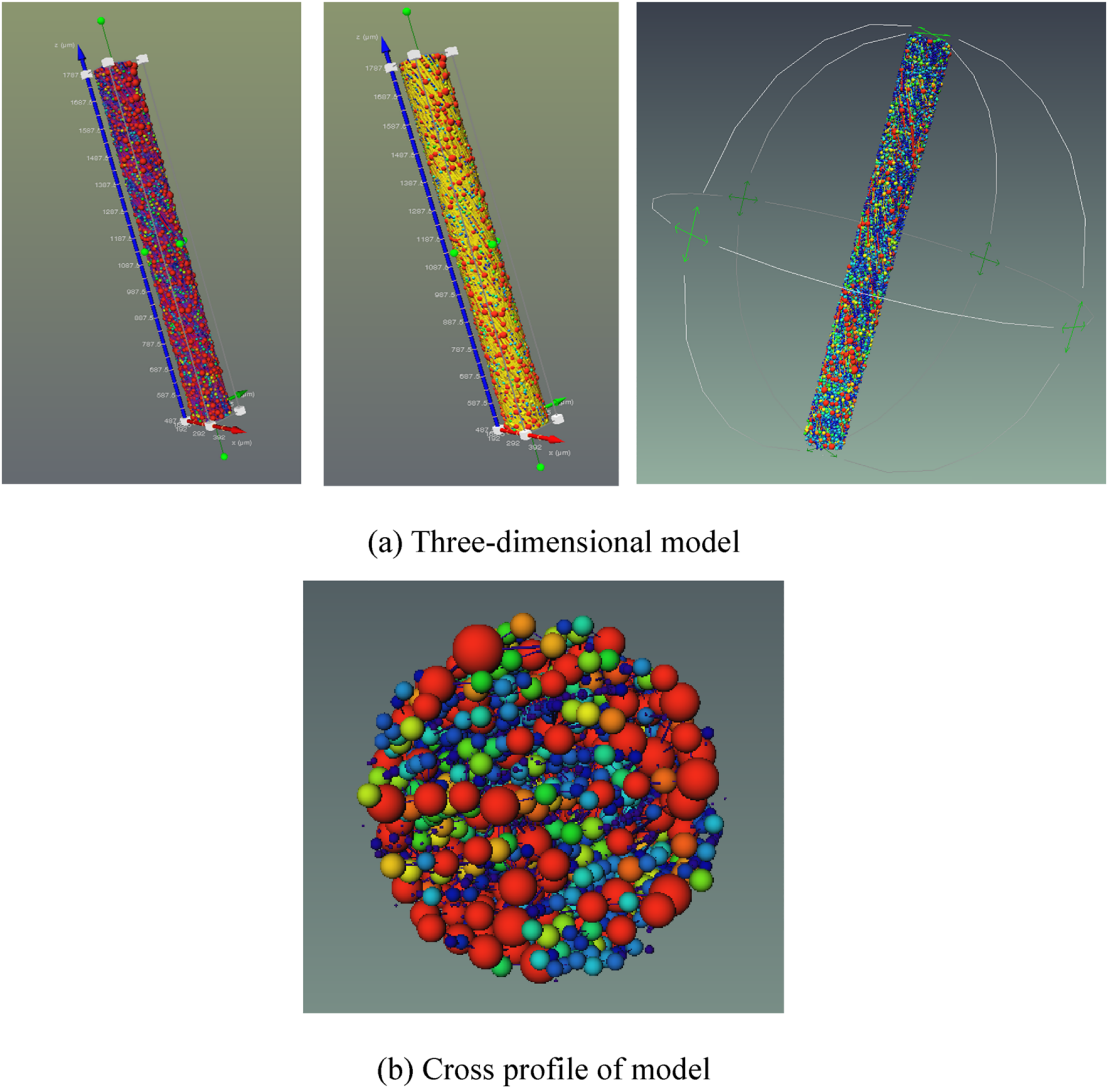
The bat model of nylon material.

In order to study the characteristics of internal pore structure of nylon more precisely, it is particularly vital to extract the corresponding pore network model to analyze the internal structure by comparing the microscopic distribution of pore structure in nylon. Employ the “Separate Objects Module” in Avizo to separate the pores, where this module labels and masks particles from a binary image representing a group of particles. Based on the data after pore separation, “Generate Pore Network Model Module” is utilized to generate the bat model. The module parameters of “Separate Objects” and “Generate Pore Network” are determined and illustrated in Table 4. On the basis of selecting the threshold value of pore structure model, the nylon pore network model was obtained after proper processing, as shown in Fig. 10. In this figure, the sphere represents the pore inside the nylon, and the cylinder represents the internal throat. The larger the sphere is, the larger the pore size is. Similarly, the thicker the cylinder is, the larger the throat radius is. The pore network model (i.e. the bat model) can directly reflect the connectivity characteristics of the pores in nylon sample.

**Table 4:** The module parameters employed in “Separate Objects” and “Generate Pore Network”.

Separate objects		Generate pore network	
Method	Skeleton-aggressive	Pore	
Interpretation	3D	Pore scale	EqRadius
Neighborhood	26	Pore coloring	EqRadius
Marker extent	1	Throats	
Output	connected object	Throat coloring	ChannelLength
Algorithm mode	repeatable	Throat scale	ChannelLength

Depending on both the pore network model^20,21^ and Avizo statistical method, the characteristic parameters of pore-fracture structure were quantitatively counted. The total number of pore reaches 15,849, and the number of throat yields 24,207. The specific microscopic parameters are shown in Table 5.

**Table 5:** Quantitative parameter statistics of pore network model in nylon sample.

Parameters	Pore radius (μm)	Pore volume (μm^3^	Pore surface area (μm^2^)	Throat radius (μm)	Throat length (μm)	Throat surface area (μm^2^)
Maximum	15.1137	14,461	6439.04	11.2059	183.189	394.495
Average	3.00549	281.00833	254.37886	2.04103	13.04778	16.33699
Minimum	0.62035	1	3.00419	0.265951	0.83474	0.222205

**Figure 11:**
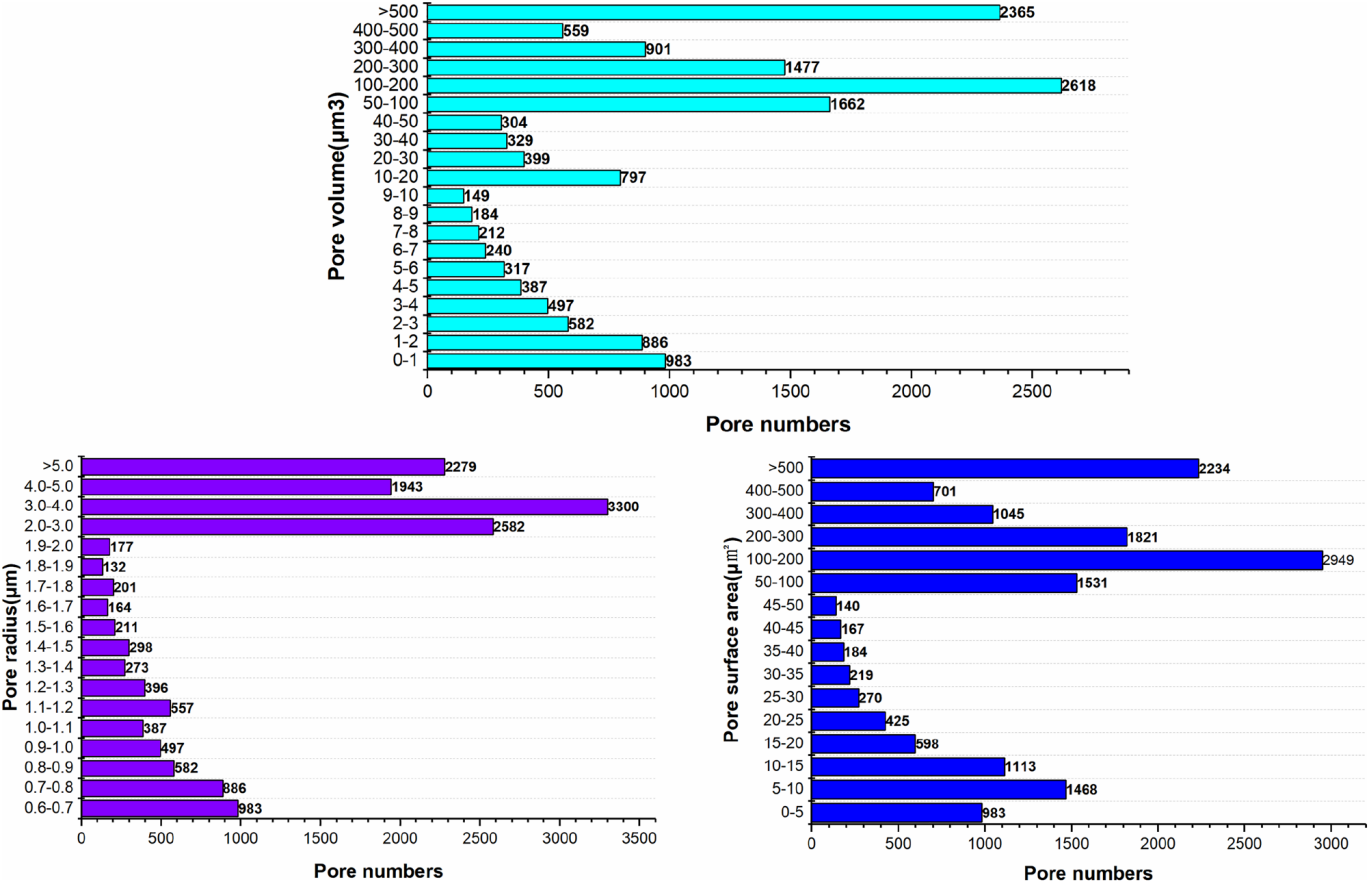
Statistics of pore quantitative parameters in pore network model.

In order to further classify the pore-fracture structure of nylon, quantitative statistical parameters of nylon structure are illustrated in Fig. 11. As can be seen from Fig. 11, the number of pores is the largest when the pore radius is in the range of 3.0–4.0 μm, accounting for 20.8% of the total. Pore volume can reflect the uniformity of the pores in nylon. The larger the pore radius is, the larger the pore volume is. Therefore, the majority of pore volumes are concentrated in the range of 100-200μm^3^, and the distribution is relatively uniform.

**Figure 12:**
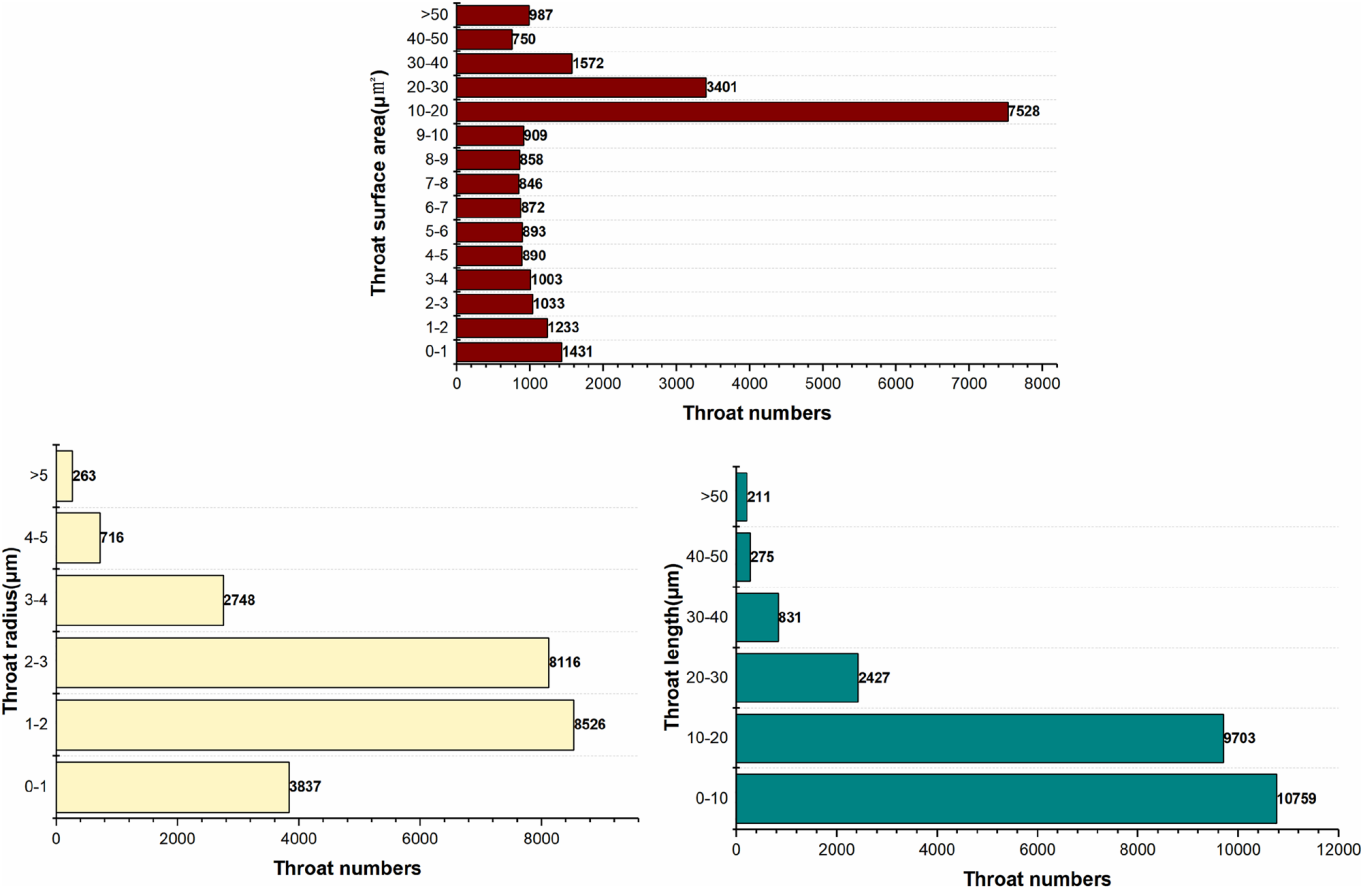
Throat quantitative parameter statistics of pore network model.

Throat is the main channel for rubber flowing through nylon. The characteristic parameters such as throat radius and length directly affect the seepage pressure and flow rate, which is illustrated as Fig. 12. Most of the throat radius are in the range of 1-2 μm, accounting for 33.9% of the total. The 12.7% throat length of nylon remains in the range of 0–10 μm, responging to the largest number of throat surface area at 0–1 μm^2^.

## Quantitative characterization of pore-fracture structure in nylon-uncured rubber composite

### CT scan

Similarly in the above part, three-dimensional non-destructive high resolution X-ray and three-dimensional microscope (nanoVoxel-3000) is also utilized to characterize the internal pore structure of nylon-uncured rubber in three-dimensional space on the microscopic scale. Avizo software is combined to realize the statistical calculation of three-dimensional connectivity, porosity, throat size and various seepage characteristic parameters of the internal throat. The responding setting of CT scanning parameters is shown in Table 6.

**Table 6:** Scanning parameters of X-ray 3D microscope.

Detector type	SOD (MM)	Voltage (kV)	Current (µA)	Scanning frame number time (s)	Time (h)	Exposure time (s)	Penetration rate (%)	SDD (MM)
Tablet 2940*2304	5.6364	80	60	1440	2	0.1	80	145.5518

**Figure 13:**
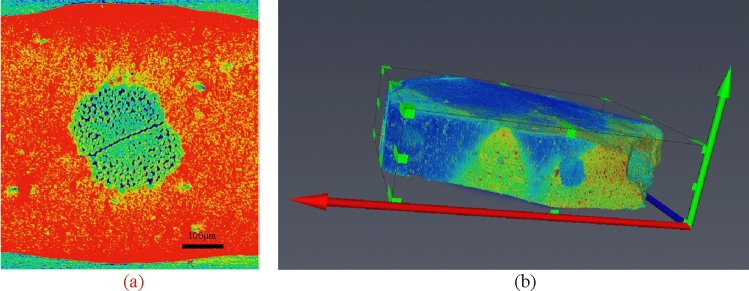
CT image of uncured nylon-rubber composite.

The section obtained after scanning of nylon-rubber composite with a size of 2772 × 787 × 2238 is presented in Fig. 13a, and its three-dimensional image is described in Fig. 13b.

### CT three-dimensional reconstruction

**Figure 14:**
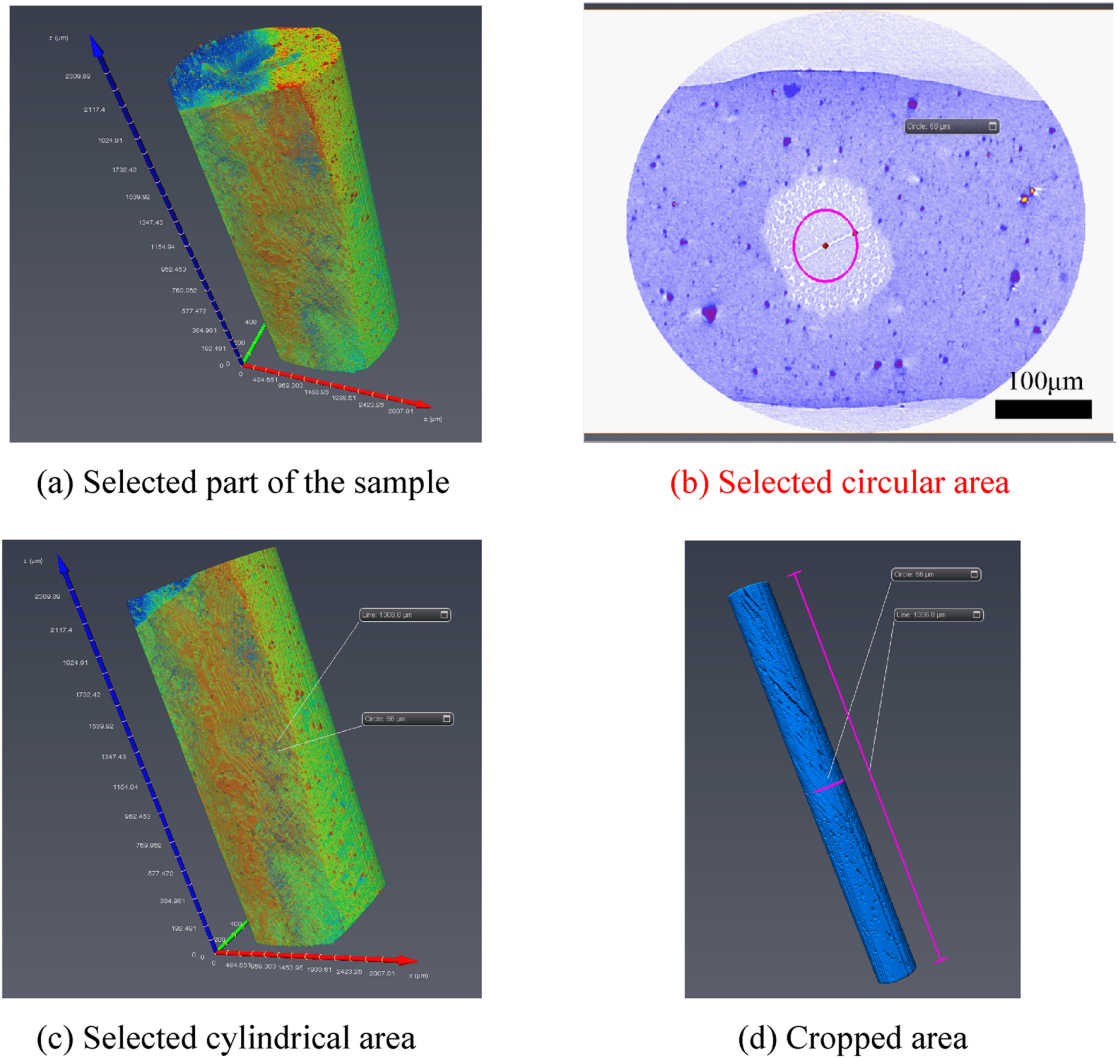
Area selection from the nylon-rubber CT images.

**Figure 15:**
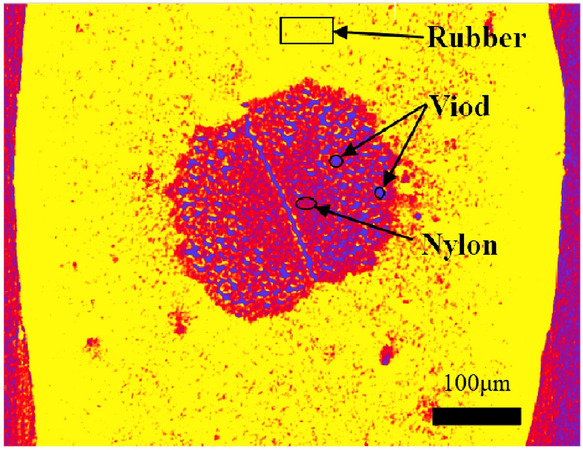
The division diagram of nylon-rubber-void (the surrounding yellow areas are rubber, red areas are nylon, and purple areas are pores).

In the construction process of pore-fracture structure model, considering the limitations of computer's storage capacity, calculation rate and other hardware conditions, the whole construction of selected regional data will lead to the collapse of computer due to huge computation. Therefore, a circular slice is adopted from the middle part of the scanned image utilized for 3D reconstruction. The center point of a nylon-rubber slice was employed as a cylinder frame with a radius of 66 μm and a height of 1306.8 μm, as shown in Fig. 14. Figure 14a shows the part of a sample cut from nylon-rubber composite, and Fig. 14b indicates the circular area selected from the slice. Figure 14c describes the pattern cutting with the cylinder. Figure 14d shows the enlarged area after cutting. Besides, the differentiation diagram of pore, nylon and rubber is shown in Fig. 15.

### Construction of pore-fracture structure model

**Figure 16:**
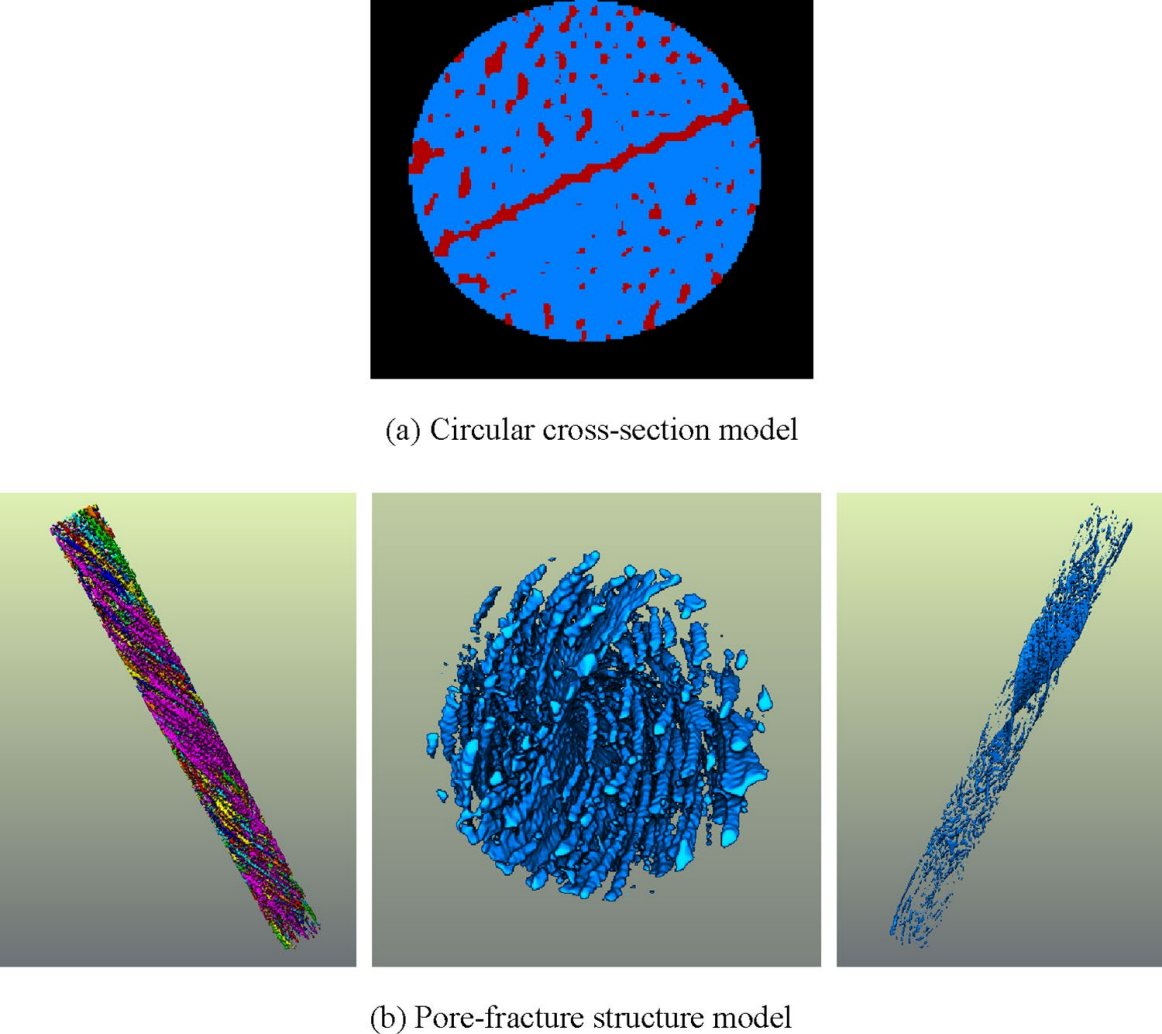
Three-dimensional distribution of the nylon-uncured rubber model.

On the basis of the three-dimensional entity obtained by threshold segmentation, the pore and fracture structure should be extracted, as demonstrated in Fig. 16a. In the process of constructing the pore-fracture structure model, it is necessary to select the threshold value of the pore-fracture structure, and the overall porosity selected should not be too small to ensure that the unconnected pores are eliminated, and the effective porosity is in line with the true porosity of nylon-rubber composite. Figure 16b shows the pore-fracture structure model after segmentation. Through the analysis of the pore-fracture structure model, the porosity of nylon-uncured rubber sample is 0.103143, and the quantitative parameters of pore model are obtained, as shown in Table 7.

**Table 7:** Quantitative parameter statistics of pore model in nylon-uncured rubber composite.

Parameters	Pore radius (μm)	Pore volume (μm^3^)	Pore surface area (μm^2^)
Maximum	61.387	968,994	710,115
Average	1.20216	114.015	107.15
Minimum	0.62035	1	3.00419

**Figure 17:**
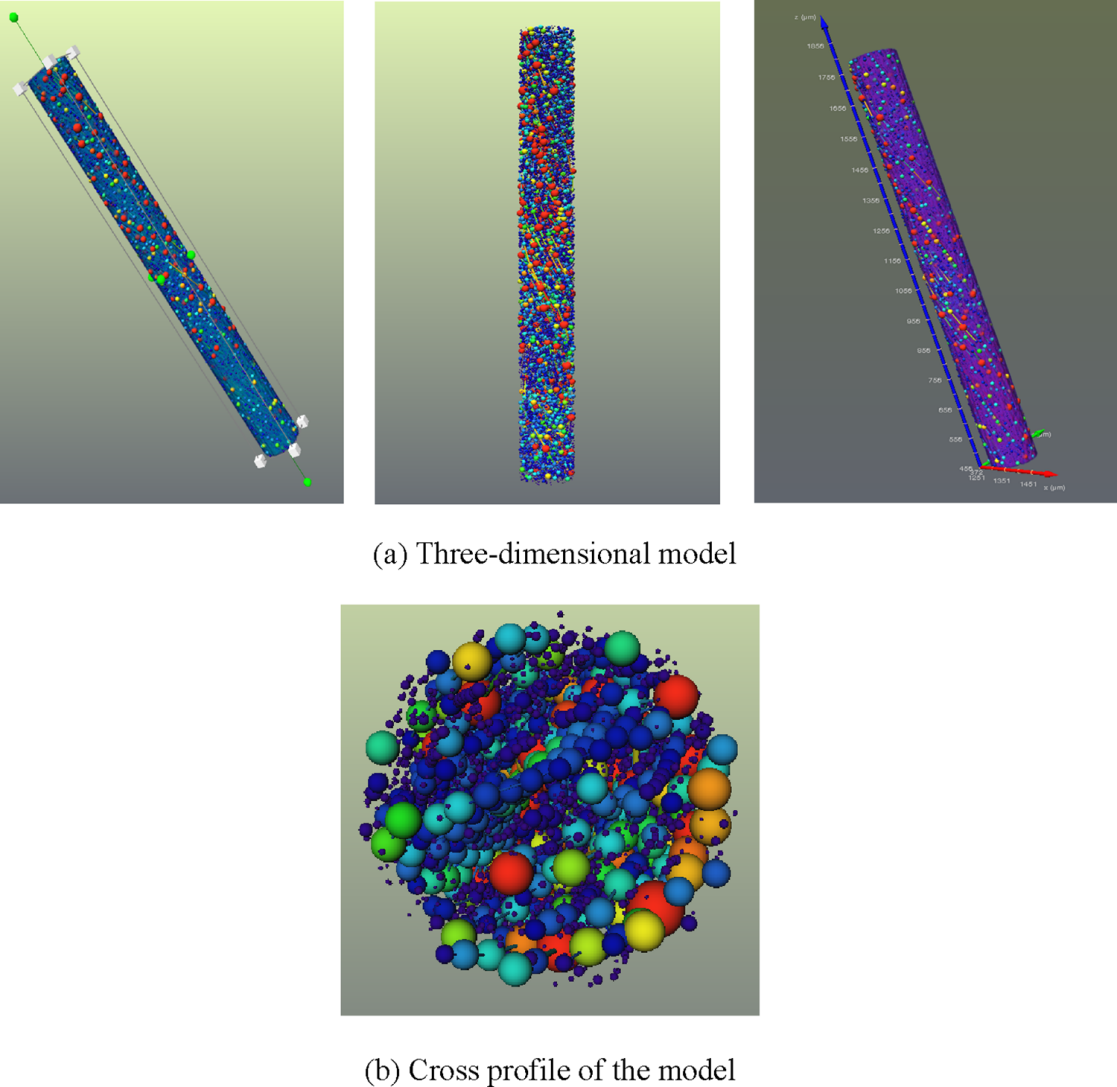
Nylon-rubber pore network model.

In order to study the internal pore structure characteristics of nylon-rubber in detail, it is particularly important to extract the corresponding pore network model to analyze the internal structure. On the basis of the selection of the threshold value of the pore structure model, the nylon-rubber pore network model was obtained after proper treatment. Figure 17 illustrates the pore network model of nylon-rubber composite. The characteristic parameters of pore and fracture structure were quantitatively counted. The total number of pore is 21,833, and the throat number is 9,189. The specific microscopic parameters are listed in Table 8.

**Table 8:** Quantitative parameter statistics of pore network model in nylon-rubber sample.

Parameters	Pore radius (μm)	Pore volume (μm^3^)	Pore surface area (μm^2^)	Throat radius (μm)	Throat length (μm)	Throat surface area (μm^2^)
Maximum	11.661	6642	5700.08	7.4056	88.0332	172.294
Average	1.63971	88.84637	92.19810	1.69683	11.04084	12.87119
Minimum	0.62035	1	3.00419	0.265957	1.00002	0.222215

**Figure 18:**
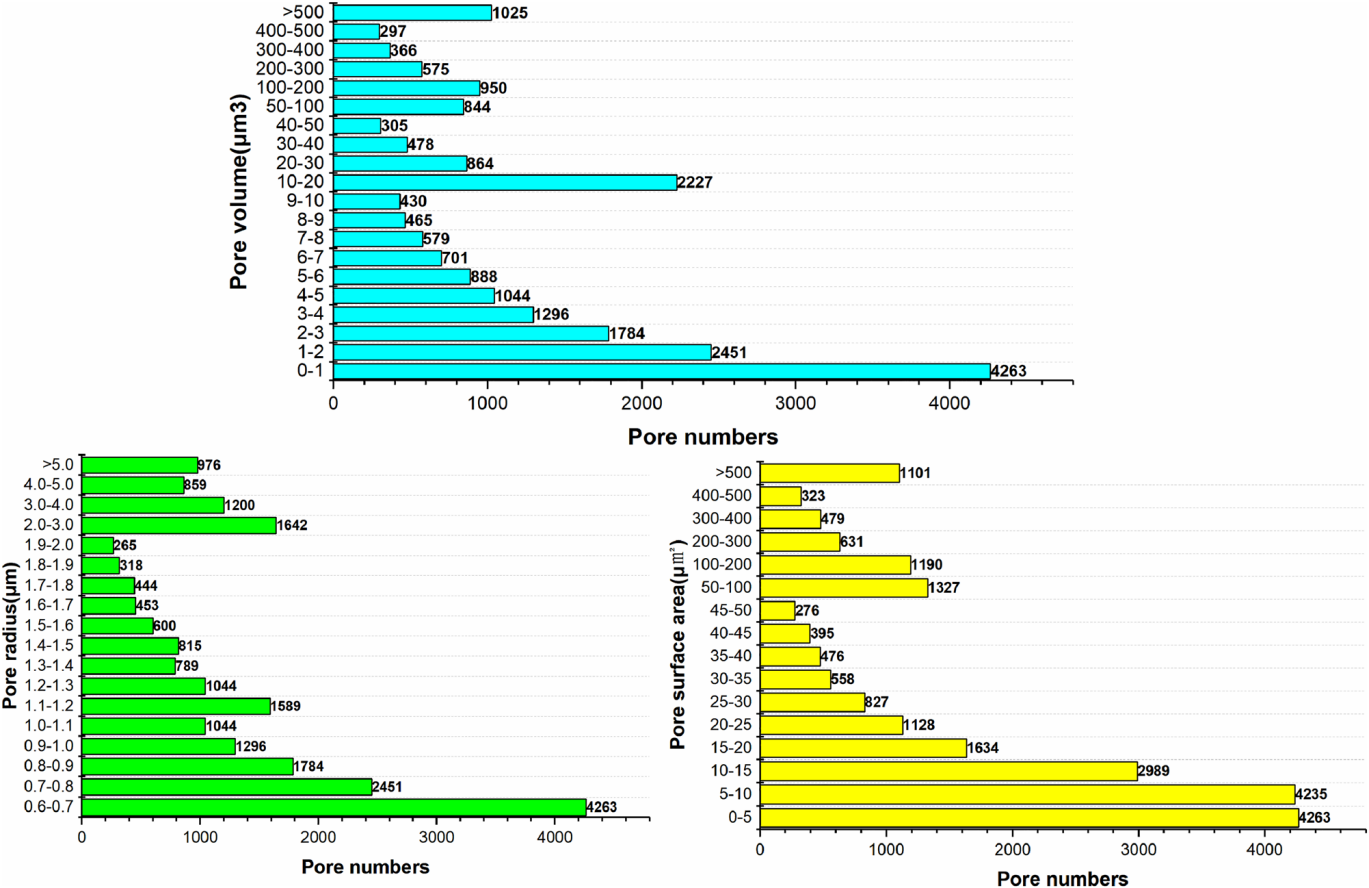
Statistics of pore quantitative parameters of nylon-uncured rubber sample.

**Figure 19:**
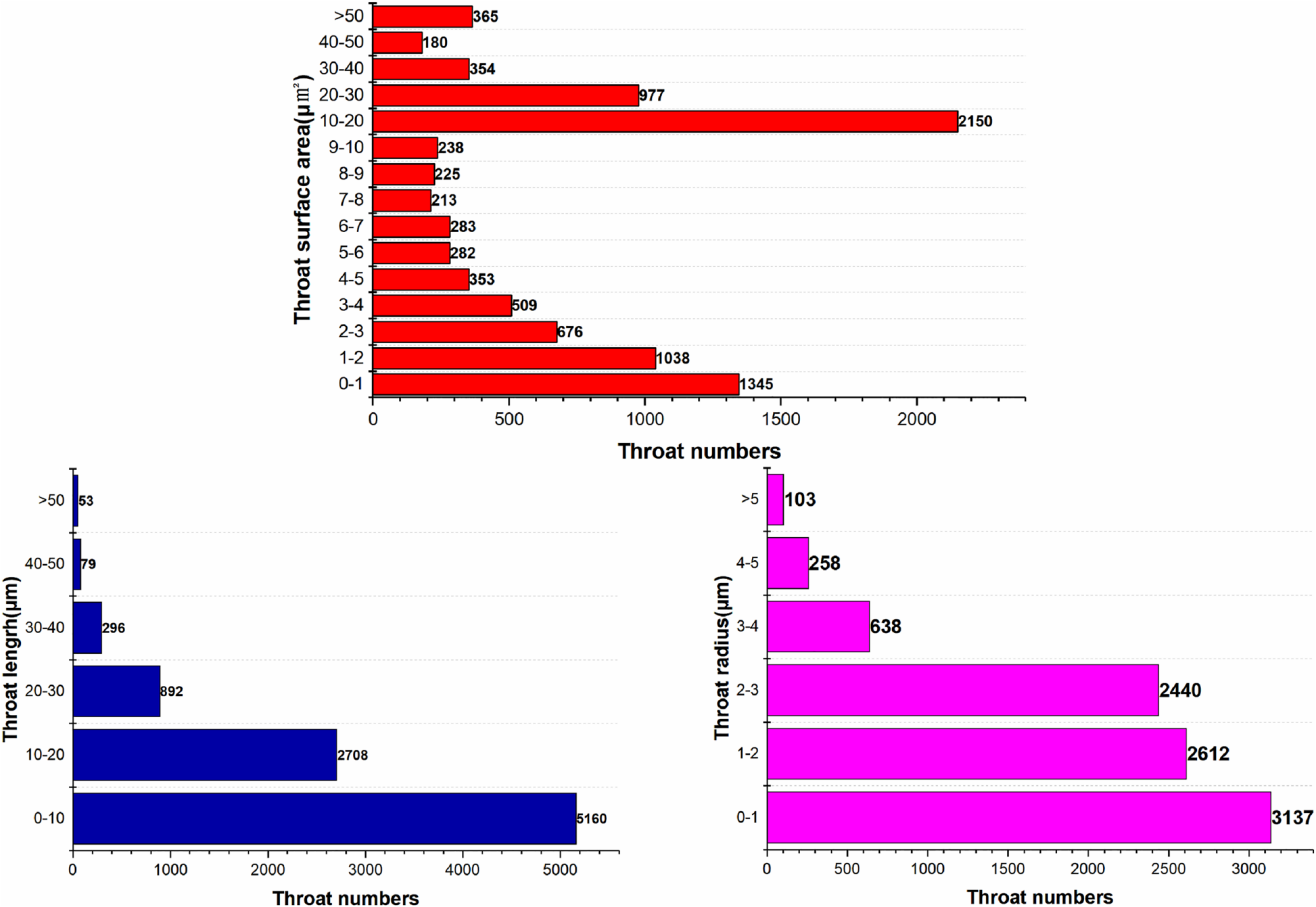
Statistics of throat quantitative parameters of nylon-uncured rubber sample.

In order to further classify the pore-fracture structure of nylon-rubber, the quantitative statistics of structural parameters in composite material are indicated in Figs. 18 and 19. Figure 18 presents that the pore number is the highest when the pore radius remains in the range from 0.6 to 0.7 μm, which occupies 19.5% of the total. Besides, pore radius curve seems decreasing linely when pore radius ranges from 0.6 to 1.1 μm. The majority of pore volume is concentrated in the range of 0–1 μm^3^, and the distribution is relatively uniform. In addition, as shown in Fig. 19, the majority of throat radius are concentrated in the range of 0–1 μm, accounting for 34.1% of the total. The throat length of 56.1% of composite is in the range of 0–10 μm.

## Approach verification

To further validate the reliability and universality of this approach, we adopt and test the other nylon-uncured rubber composite with different specification compared with that one above-mentiond. Similarly in the above parts, three-dimensional non-destructive high resolution X-ray and three-dimensional microscope (nanoVoxel-3000) is also utilized to characterize the internal pore structure of nylon-uncured rubber in three-dimensional space on the microscopic scale. Avizo software is combined to realize the statistical calculation of three-dimensional connectivity, porosity, throat size and various seepage characteristic parameters of the internal throat.

### CT scan

**Figure 20:**
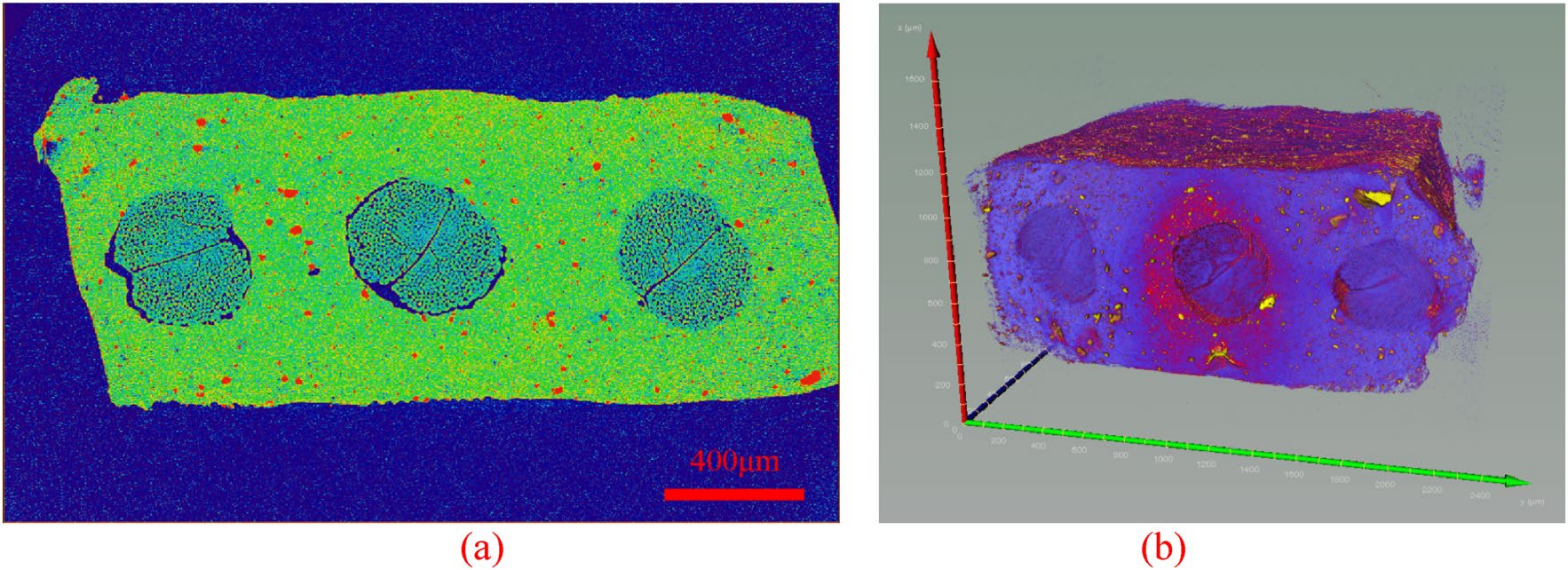
CT image of uncured nylon-rubber composite.

The section obtained after scanning of nylon-rubber composite with a size of 1600 × 2400 × 2200(μm) is presented in Fig. 20a, and its three-dimensional image is described in Fig. 20b.

### CT three-dimensional reconstruction

**Figure 21:**
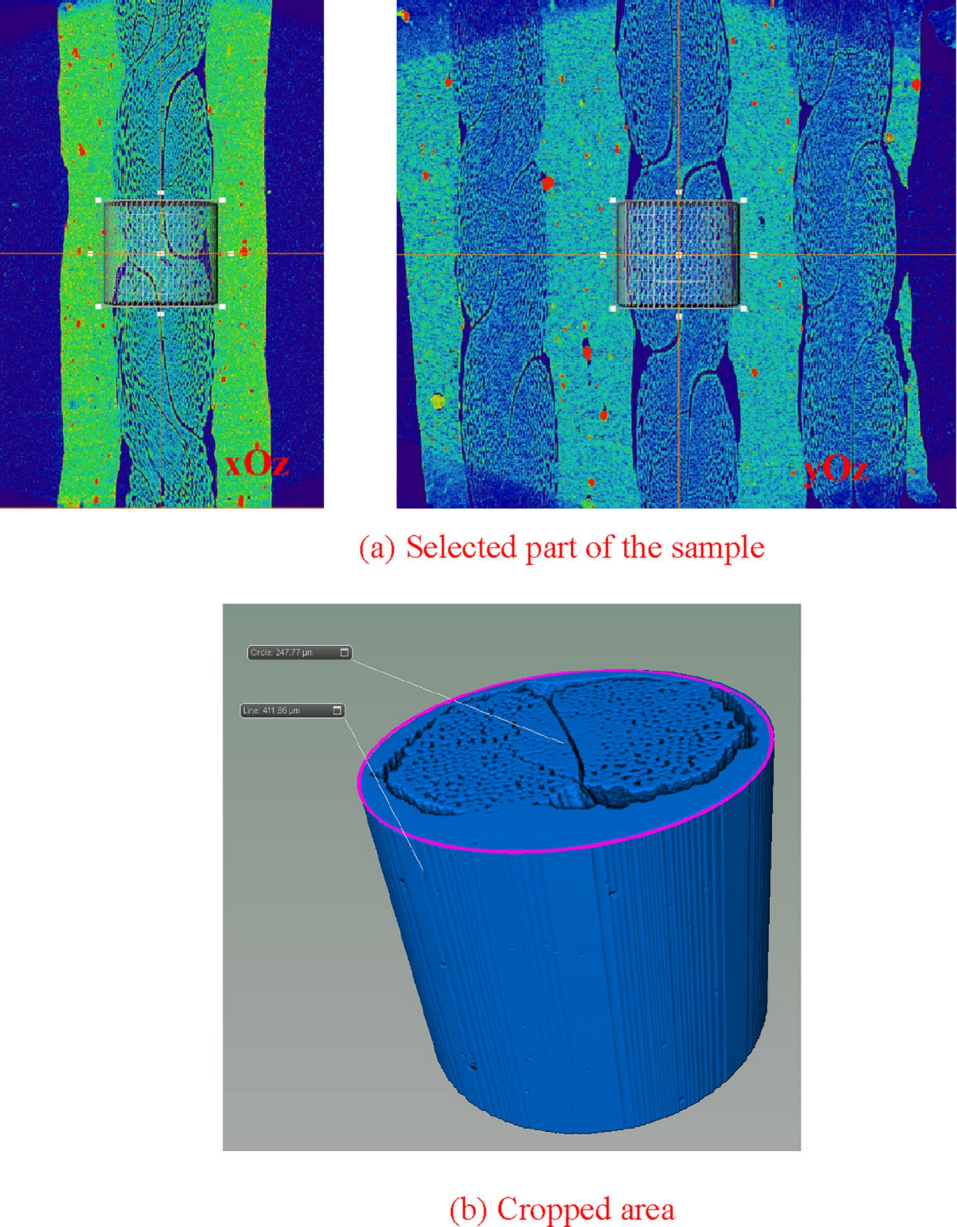
Area selection from the nylon-rubber CT images.

**Figure 22:**
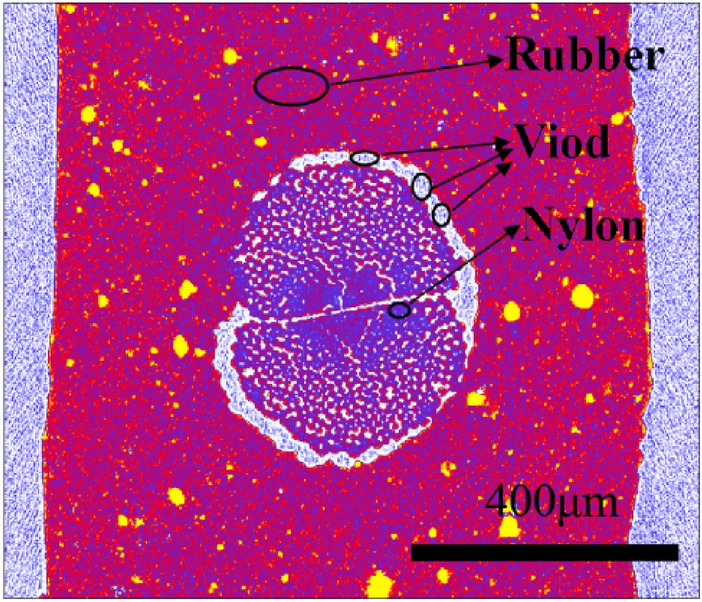
The division diagram of nylon-rubber-void (the surrounding red areas are rubber, purple areas are nylon, and white areas are pores).

In the construction process of pore-fracture structure model, considering the limitations of computer's storage capacity, calculation rate and other hardware conditions, the whole construction of selected regional data will lead to the collapse of computer due to the huge computation. Therefore, a circular slice is adopted from the middle part of the scanned image utilized for 3D reconstruction. The center point of a nylon-rubber slice is employed as a cylinder frame with a radius of 247.77 μm and a height of 411.86 μm, as shown in Fig. 21. Figure 21a shows the part of a sample cut from nylon-rubber composite, and Fig. 21b indicates the enlarged area after cutting. Besides, the differentiation diagram of pore, nylon and rubber is shown in Fig. 22.

### Construction of pore-fracture structure model

**Figure 23:**
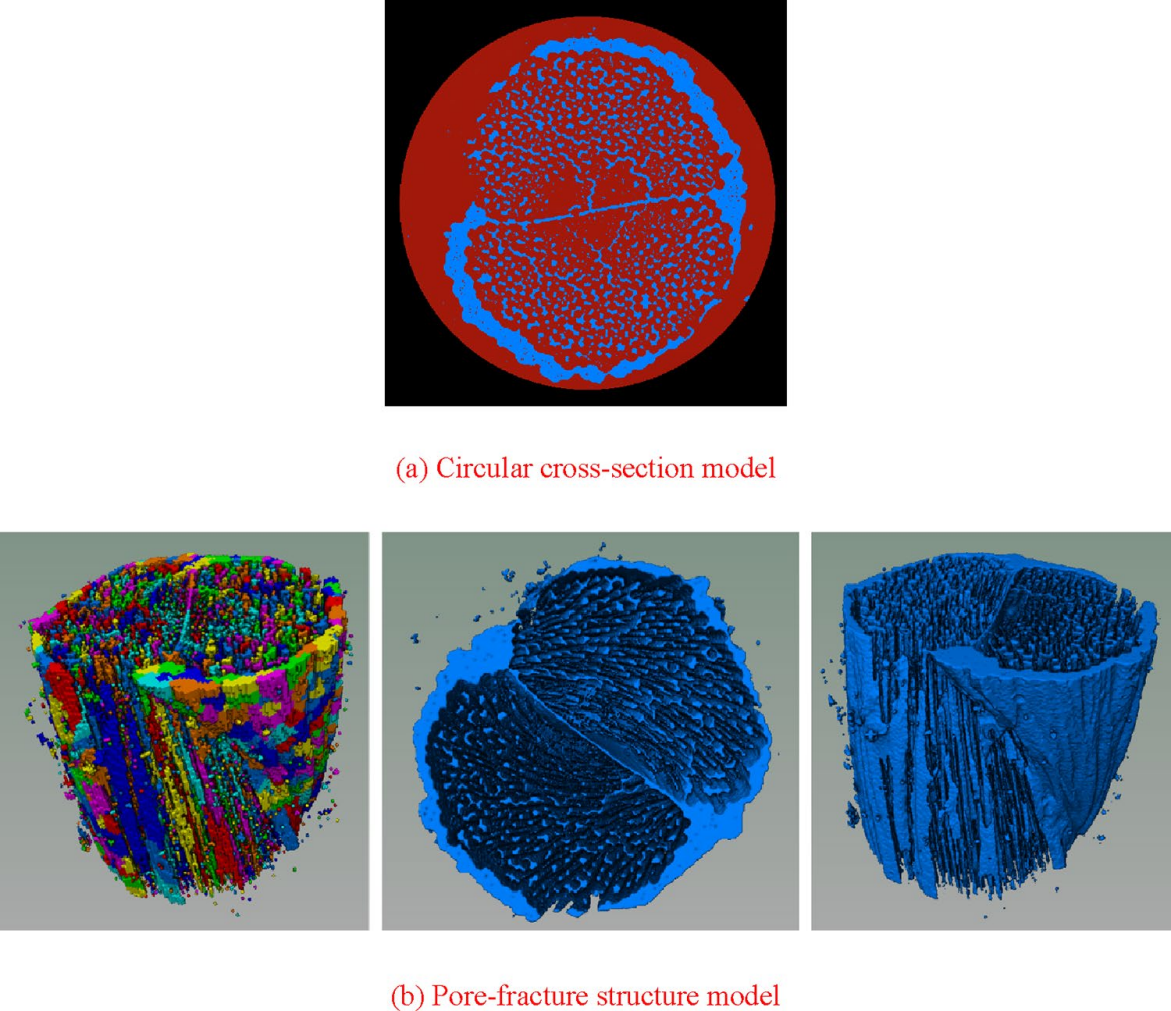
Three-dimensional distribution of the nylon-uncured rubber model.

On the basis of the three-dimensional entity obtained by threshold segmentation, the pore and fracture structure should be extracted, as demonstrated in Fig. 23a. In the process of constructing the pore-fracture structure model, it is necessary to select the threshold value of the pore-fracture structure, and the overall porosity selected should not be too small to ensure that the unconnected pores are eliminated, and the effective porosity is in line with the true porosity of nylon-rubber composite. Figure 23b shows the pore-fracture structure model after segmentation. Through the analysis of the pore-fracture structure model, the porosity of nylon-uncured rubber sample is 19.36%, and the quantitative parameters of pore model are obtained, as shown in Table 9.

**Table 9:** Quantitative parameter statistics of pore model in nylon-uncured rubber composite.

Parameters	Pore radius (μm)	Pore volume (μm^3^)	Pore surface area (μm^2^)
Maximum	163.108	18,176,700	8,699,630
Average	0.886845	271.882	141.568
Minimum	0.62035	1	3.00419

**Figure 24:**
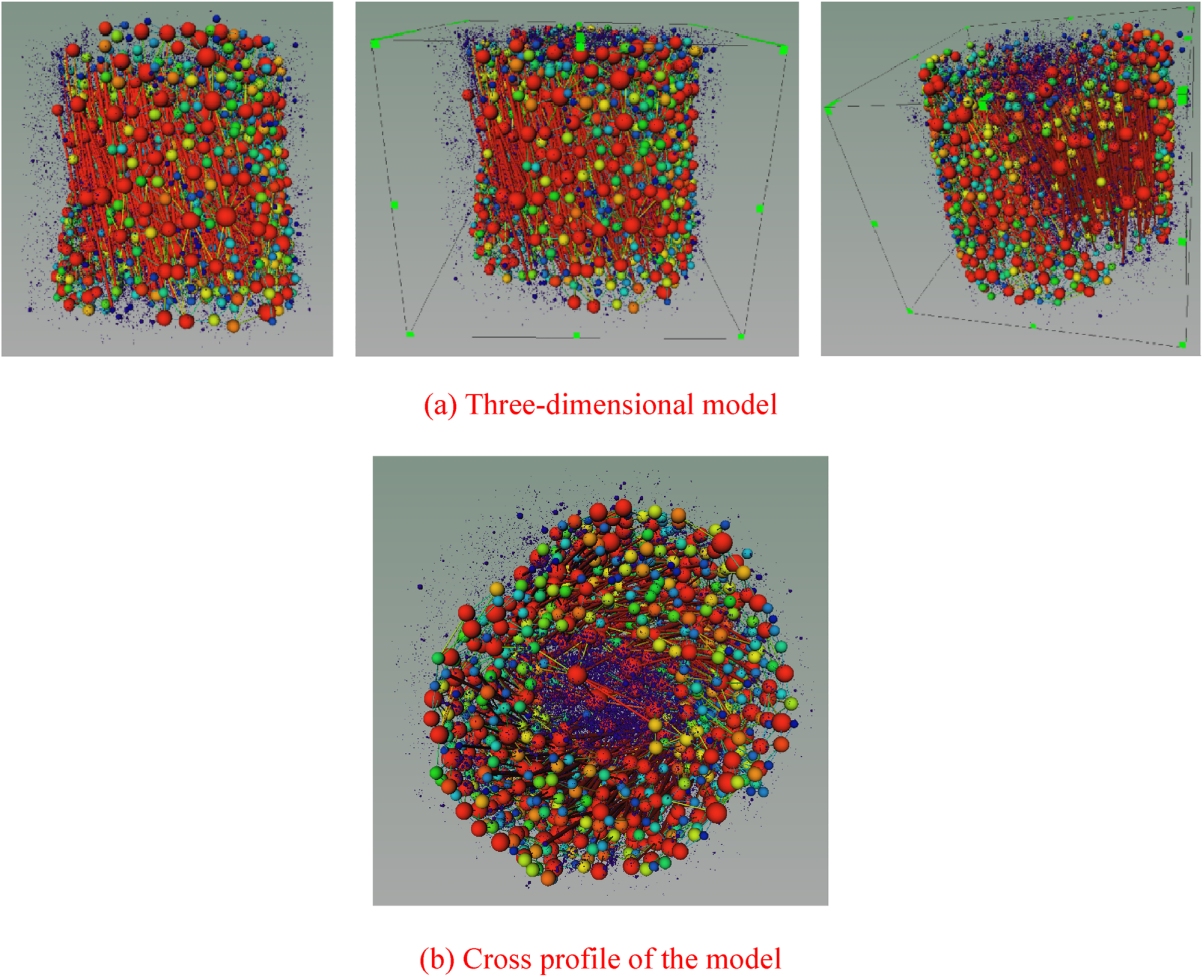
Nylon-rubber pore network model.

On the basis of the selection of the threshold value of the pore structure model, the nylon-rubber pore network model is obtained after proper treatment. Figure 24 illustrates the pore network model of nylon-rubber composite. The characteristic parameters of pore and fracture structure are quantitatively counted. The total number of pore is 65,481 and the throat number is 12,537. The specific microscopic parameters are listed in Table 10.

**Table 10:** Quantitative parameter statistics of pore network model in nylon-rubber sample.

Parameters	Pore radius (μm)	Pore volume (μm^3^)	Pore surface area (μm^2^)	Throat radius (μm)	Throat length (μm)	Throat surface area (μm^2^)
Maximum	25.4744	69,247	58,186.3	25.1342	282.766	1984.63
Average	1.2228	286.7940	172.6954	2.8944	24.3813	72.52286
Minimum	0.62035	1	3.00419	0.265945	1.02738	0.222195

**Figure 25:**
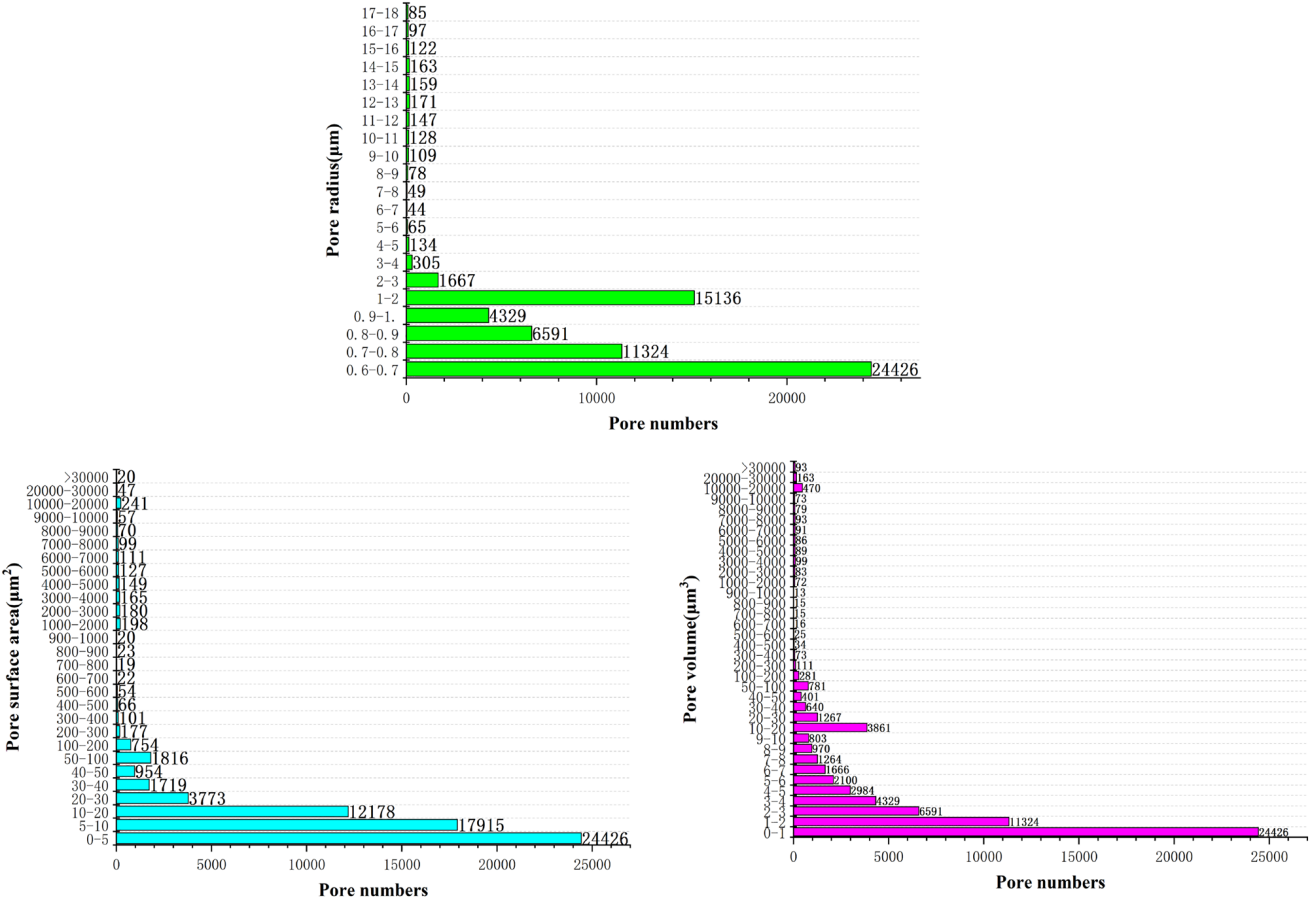
Statistics of pore quantitative parameters of nylon-uncured rubber sample.

**Figure 26:**
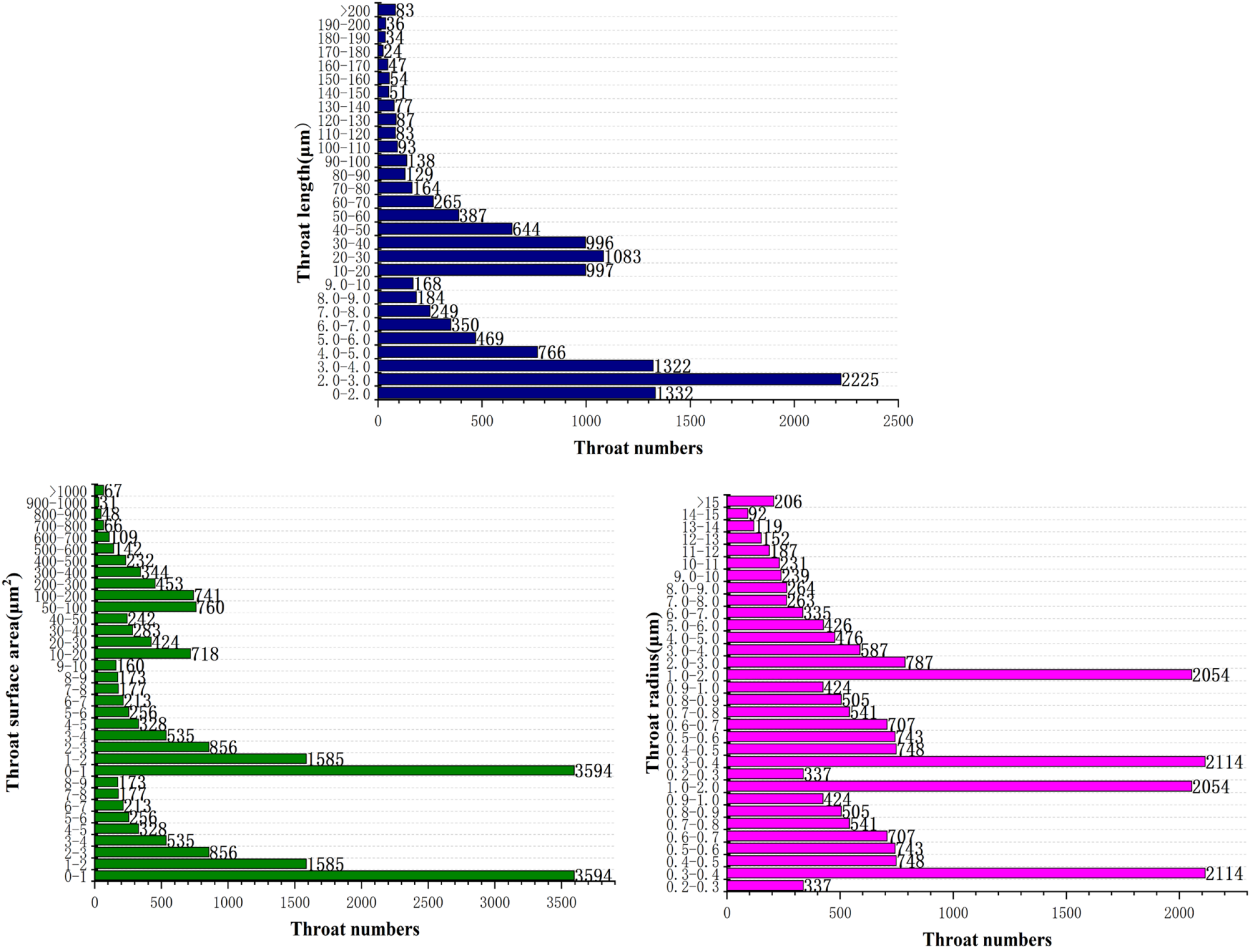
Statistics of throat quantitative parameters of nylon-uncured rubber sample.

In order to further classify the pore-fracture structure of nylon-rubber, the quantitative statistics of structural parameters in composite material are indicated in Figs.25 and 26. Figure 25 presents that the pore number is the highest when the pore radius remains in the range from 0.6 to 0.7 μm, which occupies 37.3% of the total. The majority of pore volume is concentrated in the range of 0-1μm^3^. In addition, as shown in Fig. 26, the majority of throat radius are concentrated in the range of 0–1 μm, accounting for 37.3% of the total. The histogram shows that there are 1 or 2 peaks in the pore data The throat length of 3.4% of composite is in the range of 2–3 μm. Compared with the data of throat channel volume, throat channel area, and throat channel radius, the data shows that there are 2 or 3 peaks.

## Discussions

As can be seen from Fig. 11, the pore number of nylon material is the largest when the pore radius is in the range of 3-4 μm, accounting for 20.8% of the total. However, Fig. 18 presents that the pore number of nylon-rubber composite with the pore radius 3–4 μm occupies merely 5.49% of the total. Moreover, the average pore radius of nylon is larger than that of nylon-rubber composite, which is availble to be reached by Tables 5 and 8. The pore surface area and pore volume have the perfect consistent pattern with that of pore radius.

On the other hand, as can be acquired from Fig. 12, the throat number of nylon material is the largest when the throat radius is in the range of 1–2 μm, accounting for 33.9% of the total. However, Fig. 19 illustrates that the throat number of nylon-rubber composite with 0–1 μm throat radius occupies the majority of the total, which reaches up to 34.1%. Furthermore, the average throat radius of nylon is far larger than that of nylon-rubber composite, which is explicitly realized from Tables 5 and 8. The throat surface area and throat length as well have the perfect consistent pattern with that of throat radius.

Above mentioned phenomenon can be explained by the discrepancy of gas seepage capability between the solid (nylon) one-phase and solid (nylon)—viscoelastic body (rubber) two-phases. The gap between nylon arrangement and the fine structure inside nylon can be equivalent to the double medium of fractures and pores. The double medium seepage method should be deeply paid attention and employed in future researches to clarify the penetration of rubber into nylon.

## Conclusions

In this paper, the X-ray three-dimensional (3D) microscope is applied for the nylon material and nylon-uncured rubber composite, respectively. By employing the Avizo software, three dimensional reconstruction is realized and pore-fracture network model is set up by intercepting the part of area. Furthermore, the quantitative statistics and comparative analysis is carried out to clarify the distribution law of pore-fracture of the solid (nylon)-gas (pore) two-phases with that of solid (nylon)-viscoelastic body (rubber)-gas (pore) three-phases composite, mainly including the numbers of pore/throat, as well as microscopic pore-fracture connectivity, etc.

1. Quantitative analysis of nylon showed that the pore number of nylon material is the largest when the pore radius is in the range of 3–4 μm, accounting for 20.8% of the total. However, the pore number of nylon-rubber composite with the pore radius 3–4 μm occupies merely 5.49% of the total.
2. The establishment of pore network model can be more intuitive to observe the distribution of pore, the average pore radius of nylon is larger than that of nylon-rubber composite, and the pore surface area and pore volume have the perfect consistent pattern with that of pore radius.
3. The throat number of nylon material is the largest when the throat radius is in the range of 1–2 μm, accounting for 33.9% of the total. However, the throat number of nylon-rubber composite with 0–1 μm throat radius occupies the majority of the total, which reaches up to 34.1%.
4. The average throat radius of nylon is far larger than that of nylon-rubber composite. The throat surface area and throat length as well have the perfect consistent pattern with that of throat radius, which phenomenon can be explained by the discrepancy of gas seepage capability between the solid (nylon) one-phase and solid (nylon)—viscoelastic body (rubber) two-phases.
5. The gas seepage capability in solid (nylon) phase presents the obvious superiority than that in solid (nylon)-viscoelastic body (rubber) phases.
